# Pediatric liver transplant for maple syrup urine disease a single center experience

**DOI:** 10.3389/fped.2025.1724099

**Published:** 2025-12-10

**Authors:** Ibrahim Hassan, Samah Mahjoub, Bushra Jalodi, Iftikhar Khan, Ahmed Zidan, Mansour Tawfeeq, Mohammed Al Qahtani, Razan Bader, Eyad Gadour

**Affiliations:** 1 Multiorgan Transplant Centre of Excellence, Liver Transplantation Department, King Fahad Specialist Hospital, Dammam, Saudi Arabia; 2 Department of Surgery, Assiut University Hospital, Assiut, Egypt; 3 Department of Surgery, Imam Abdulrahman Bin Faisal University, Dammam, Saudi Arabia; 4 Internal Medicine Department, School of Medicine, Zamzam University College, Khartoum, Sudan

**Keywords:** maple syrup urine disease (MSUD), liver transplant, pediatrics, graft survival, branched-chain alpha-ketoacid dehydrogenase complex

## Abstract

**Introduction:**

Maple syrup urine disease (MSUD) is an autosomal recessive inborn error of branched-chain amino acid metabolism caused by an inherited deficiency of branched-chain alpha-ketoacid dehydrogenase (BCKDH) activity that degrades isoleucine, leucine, and valine. Liver transplantation (LT) is a therapeutic option to treat the classical severe type of MSUD. This study aimed to assess and compare liver biochemical outcomes and amino BCAAs levels post-LT according to different types of donors.

**Methods:**

Retrospective cohort analysis of 10 patient medical records diagnosed as MSUD who underwent LT in King Fahad Specialist Hospital-Dammam between January 2013 and May 2023.

**Results:**

Ten pediatric patients diagnosed with MSUD who had finished 1 year of follow-up after LT were included in the study. The median age of diagnosis among the pediatric patients was 1 month (as it is included in the national neonatal screening). Besides, the median age for LT was 123 months (10 years 3 months) (with a range of 9–173 months). Availability of milk formula, prevention of further neurological insult, and difficulty in controlling the protein intake were the main indications for LT. Post LT, six patients (60%) were immediately initiated on a regular diet, and four patients (40%) waited for 3 months before starting a regular diet. All the patients were on Tacrolimus as immunosuppression, with three patients started on Mycophenolate because of biopsy-proven acute cellular rejection. Our result showed 100% 1-year graft survival and 100% until the time of report, with no donor complications. 70% of the explanted liver had been re-transplanted as a domino liver transplant. No clinical or laboratory difference when comparing living-related to living-unrelated or deceased donor liver transplant. 100% of our patients and families showed satisfaction with the decision of liver transplant.

**Conclusion:**

MSUD post liver transplant showed a 100% graft survival rate, and all patients and families showed satisfaction. In settings with limited deceased donor organs, living-related donor LT (even from heterozygous carriers) is a viable option for MSUD, yielding comparable metabolic control and graft outcomes to other donor types.

## Introduction

Maple syrup urine disease (MSUD) is an autosomal recessive inborn error of metabolism, resulting from a genetic deficiency in the branched-chain alpha-ketoacid dehydrogenase (BCKDH) activity, which is crucial for the branched-chain amino acids (BCAAs) isoleucine, leucine, and valine. The accumulation of these BCAAs can lead to ketoacidosis and severe neurological damage, potentially resulting in death if untreated (1–3). Typically, MSUD presents as a neonatal encephalopathy, which can be fatal without prompt and aggressive treatment. Even with strict dietary management, some patients experience recurrent, life-threatening metabolic crises during periods of catabolic stress, leading to significant neurocognitive decline. Liver transplantation (LT) is considered a treatment option for severe cases of MSUD. Although the liver only contributes 9%–13% of the body's total BCKDH activity (4), LT is often sufficient to cure MSUD and eliminate the necessity for dietary protein restriction (2, 5). Literature evidence has shown that LT effectively regulates BCAA metabolism and prevents further neurological damage (5). Following LT, patients with MSUD typically achieve normal BCAA levels and halt the progression of neurological damage, although existing damage cannot be reversed (2). Most patients experience a rapid decrease in leucine levels to normal post-LT, with BCAA levels remaining stable over long-term follow-up (5).

From the above literature evidence, LT remains the cornerstone for modern medical practice. However, long-term patient and graft survival outcomes are still impacted by post-LT metabolic adaptation and donor types. Understanding how BCAAs levels vary across deceased, living related, and leaving unrelated donor recipients may offer substantial insights into patient recovery and graft stability. With a few studies integrating clinical outcome and metabolic data in this context, our study aims to address this gap. Therefore, we strive to assess and compare liver biochemical outcomes and amino BCAAs levels post-LT according to different types of donors.

## Method

This study was designed as a single-centre retrospective cohort study, conducted at King Fahad Specialist Hospital, Dammam-Saudi Arabia between January 2013 and May 2023. The study included 10 pediatric patients (7 males) diagnosed with MSUD who underwent LT. This institution is the only center in the country under the Ministry of Health that performs liver transplants. The diagnosis of MSUD in these patients was confirmed through clinical and genetic testing. All patients were referred either from our hospital's metabolic unit or regional hospitals specifically for LT.

Before the transplant, all patients were maintained on a protein-restricted diet supplemented with valine and isoleucine under the supervision of a dietitian and a metabolic team. Among the 10 patients, one received a liver transplant from a deceased donor, four underwent transplantation using livers from unrelated living donors, and the remaining patients received transplants from related living donors. Post-transplant, a standardized protocol was followed for all patients, which included induction therapy with Basiliximab and intravenous Methylprednisolone, followed by a gradual tapering of Prednisolone over 4–6 months. Patients were maintained on Tacrolimus as monotherapy or a combination of Tacrolimus and Mycophenolate in cases of repeated acute cellular rejection or when renal-sparing medication was necessary. Additionally, seven patients' explanted livers were donated to consenting adult recipients without MSUD through “domino” transplantation.

The data collected included the patients’ age at MSUD diagnosis, age at transplantation, pre-transplant and current diet, and the timeline for starting a regular diet post-transplant. Information on current immunosuppressive medications and donor types was also gathered. Biochemical data collected encompassed amino acid levels of isoleucine, leucine, and valine before the transplant and at the end of 1- and 5-year post-transplant, when applicable, corresponding to the time point at which current immunosuppression data were recorded. Other data included direct bilirubin, albumin, alanine transaminase (ALT), aspartate transaminase (AST), alkaline phosphatase, gamma-glutamyl transferase (GGT), and international normalized ratio (INR), all of which were used to assess graft outcomes at the end of 5-year follow-up.

Patient and family satisfaction post-liver transplant was evaluated by inquiring whether they were happy with the decision to proceed with the transplant or if they had any regrets.

The study protocol received ethical approval from the King Fahad Specialist Hospital Institutional Review Board (IRB) under approval number MOTC0009.

### Statistical analysis

We utilized the IBM Statistical Package for the Social Sciences (SPSS®), version 22.0 for Windows (SPSS Inc., Armonk, New York, United States) for statistical analysis. Since our sample was small with unbalanced groups, we only conducted descriptive statistics. For quantitative data, the presentation was in median and range (interquartile ranges – IQRs), whereas for qualitative data, the presentation was in frequencies/counts (*n*) and percentages (%). We did not report inferential hypothesis testing – such as ANOVA – and *p*-values since substantial comparisons among groups were not reasonable.

## Results

### Demographics and patient information

The study included 10 pediatric patients diagnosed with MSUD who completed a one-year follow-up after LT. Among these patients, 70% were male. The median age at diagnosis was 1 month, as MSUD is part of the national neonatal screening program, while the median age at the time of LT was 123 months (ranging from 9 to 173 months). The primary indications for LT were the availability of milk formula, prevention of further neurological damage, and challenges in controlling protein intake. Pre-LT, all patients adhered to a protein-restricted diet. Post-transplant, six patients (60%) immediately transitioned to a regular diet, while four patients (40%) delayed this transition for three months. All patients received Tacrolimus for immunosuppression; however, three patients were additionally administered Mycophenolate due to biopsy-confirmed acute cellular rejection. The age at the last follow-up ranged from 2 to 18 years, with a median of approximately 9 years. Table 1 details the baseline characteristics of pediatric pre- and post-LT.

**Table 1:** Pre and post-liver transplantation baseline characteristics.

Clinical Variable	Patient Data
Sex	
Male *n* (%)	7 (70%)
Age at diagnosis (month)	
Median (range)	1 (1–37)
Age at transplant (month)	
Median (range)	123 (9–173)
Indication for liver transplant availability of formula *n* (%)	6 (60%)
Prevent further neurological insult *n* (%)	7 (70%)
Difficult to control protein intake *n* (%)	7 (70%)
Current diet	
Regular diet *n* (%)	10 (100%)
Time to start regular diet *n* (percentage)	
3- 3-month post-LT	4 (40%)
Immediately post-LT	6 (60%)
Duration of follow-up	
Median (range)	3 years (1–7 years)
Current immunosuppressive medication *n* (%)	
Tacrolimus	7 (70%)
Tacrolimus Mycophenolate	3 (30%)

LT, liver transplantation.

### Changes in amino acid levels (µmol/L)

**Figure 1:**
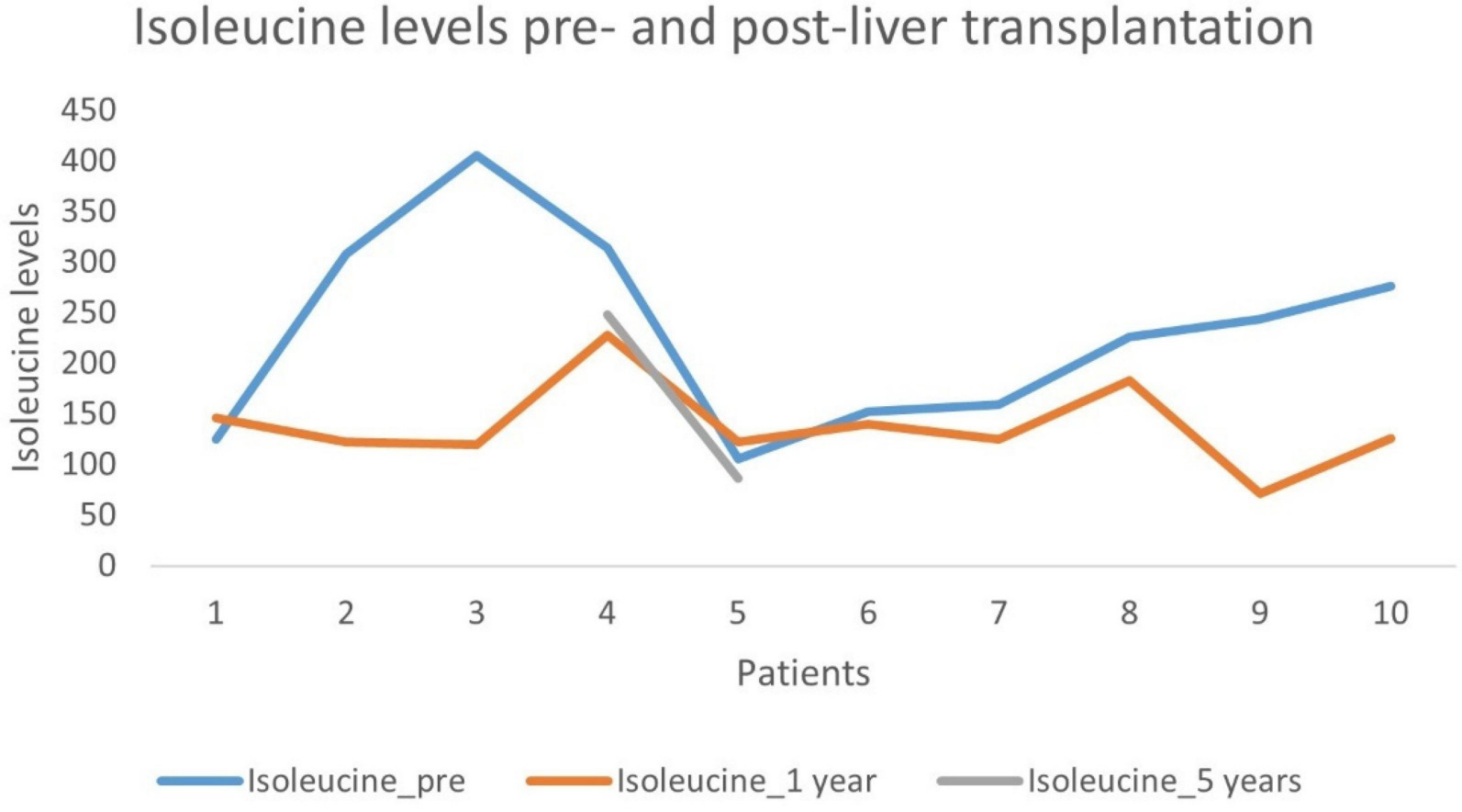
Isoleucine level pre- and post-liver transplantation.

Isoleucine: Levels ranged from 106 to 406 pre-LT, from 71 to 183 at one-year post-LT, and from 86 to 248 at five years post-LT (Figure 1).

**Figure 2:**
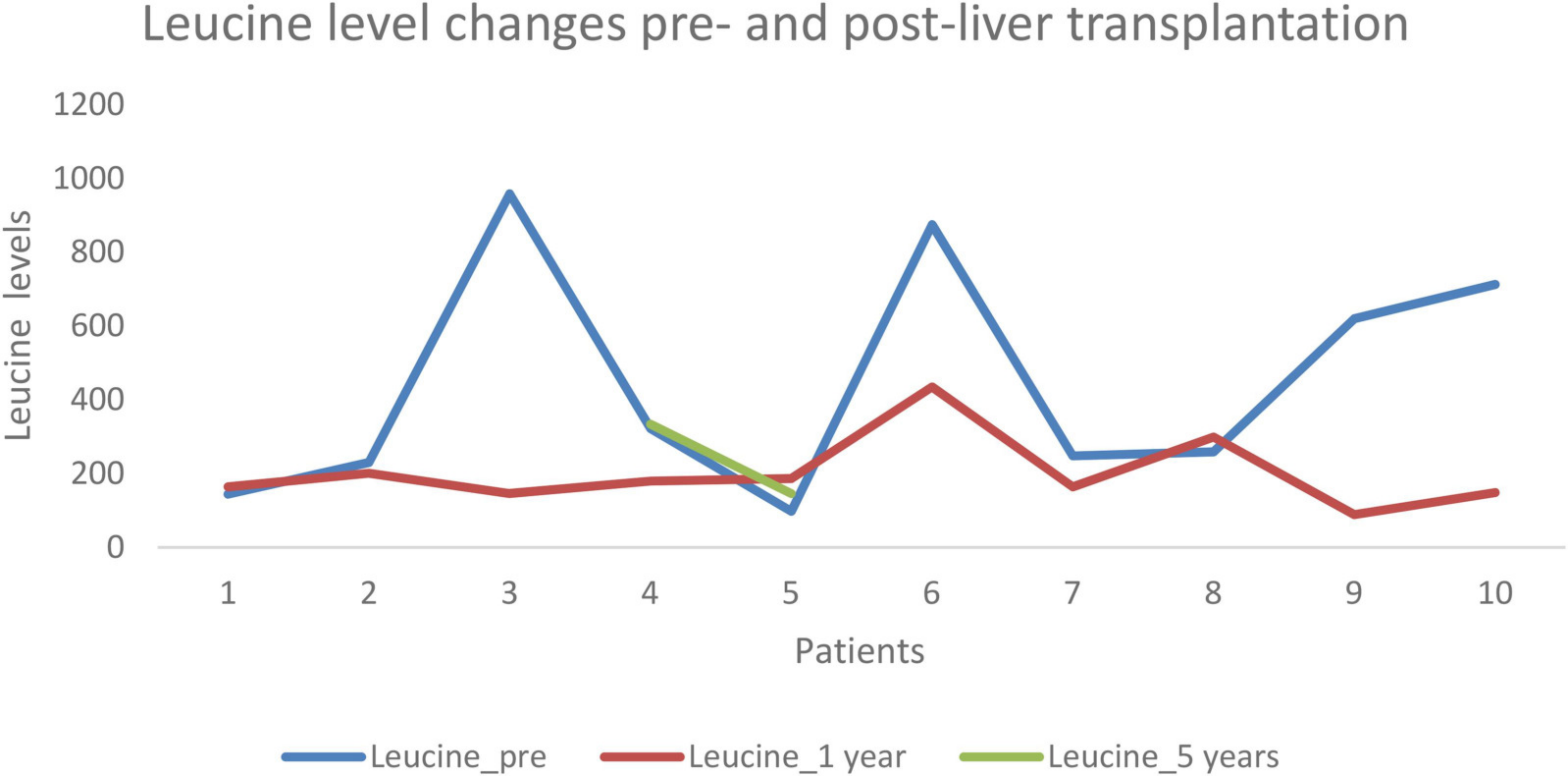
Leucine level pre- and post-liver transplantation.

Leucine: Levels ranged from 98 to 959 pre-LT, from 89 to 435 at one-year post-LT, and from 96 to 496 at five years post-transplant (Figure 2).

**Figure 3:**
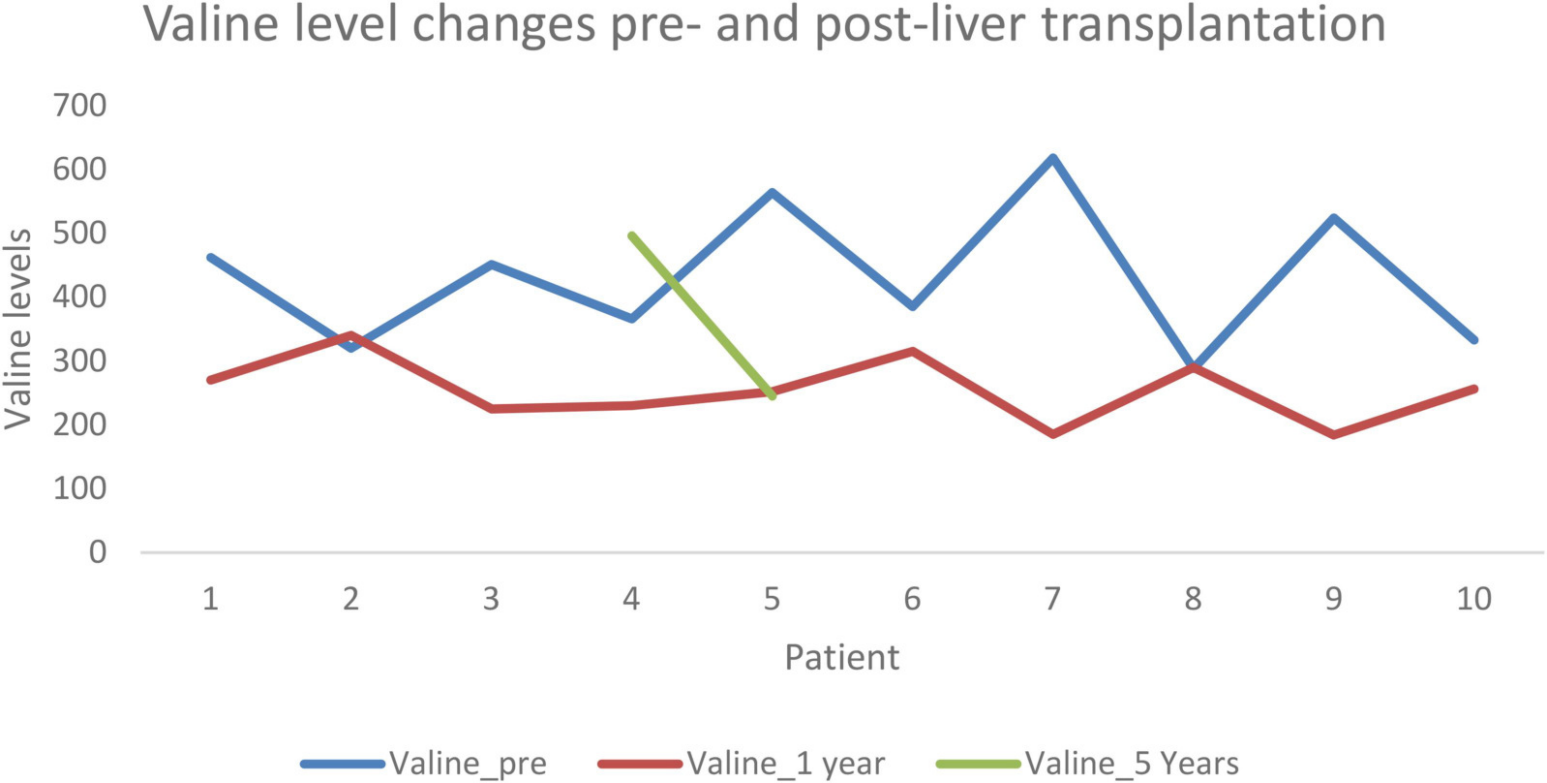
Valine level pre- and post-liver transplantation.

Valine: Levels ranged from 288 to 618 pre-LT, from 184 to 340 at one-year post-LT, and from 248 to 496 at five years post-transplant (Figure 3).

Only three patients had the record of BCAAs levels at the end of the 5-year follow-up post-LT.

### Post-transplant changes in BCAAs and graft outcomes

Table 2 details the changes in post-LT levels of BCAAs and graft outcomes among patients based on different donor groups. Among all the groups, amino acid levels were higher than normal ranges. After LT, isoleucine, leucine, and valine normalized with a median of 126.0 (121.0–143.0) µmol/L, 164 (147.5–318.0) µmol/L, and 270.0 (240.5–327.5) µmol/L, respectively, among living related donors after one-year follow-up. Isoleucine and leucine levels remained within the normal ranges at the end of 5-year follow-up (136.0 and 199.0 µmol/L, respectively), whereas valine levels increased to 353.0 µmol/L.

**Table 2:** Changes in amino acid levels and graft outcomes among different donor groups.

Donor type	Isoleucine (µmol/L) Pre-LT [median (IQR)]	Isoleucine 1-year post-LT [median (IQR)]	Isoleucine 5-year post-LT (median)	Leucine Pre-LT [median (IQR)]	Leucine 1-year post-LT [median (IQR)]	Leucine 5-year post-LT (median)	Valine Pre-LT [median (IQR)]	Valine 1-year post-LT [median (IQR)]	Valine 5-year post-LT (median)	Total Bilirubin (mg/L) [median (IQR)]	ALT (U/L) [median (IQR)]	AST (U/L) [median (IQR)]	Albumin (g/L) [median (IQR)]	GGT (U/L) [median (IQR)]
Living related	276.0 (138.5–357.0)	126.0 (121.0–143.0)	136.0	713 (187.0–917.0)	164 (147.5–318.0)	199.0	385 (326.0–456.5)	270.0 (240.5–327.5)	353.0	7.0 (5.1–7.9)	20.0 (17.5–41.5)	26.0 (19.0–50.0)	40.0 (39.5–43.0)	20.0 (12.5–32.0)
Living unrelated	192.5 (119.25–239.5)	123.5 (83.75–168.5)	86.0	253.50 (135.5–529.6)	175.5 (107.8–271.0)	146.0	544.0 (347.0–604.5)	218.5 (184.3–280.5)	245.0	8.75 (6.5–16.4)	31.5 (21.8–36.8)	36.5 (27.8–39.3)	42.5 (34.5–44.5)	18.0 (9.3–32.0)
Deceased	314.0	228.0	248.0	322.0	180.0	334.0	366.0	230.0	496.0	16.0	29.0	22.0	43.0	21.0

Among living unrelated donors, 1-year and 5-year post-LT isoleucine, leucine, and valine levels were 123.5 (83.75–168.5) µmol/L and 86.0 µmol/L, 175.5 (107.8–271.0) µmol/L and 146.0 µmol/L, and 218.5 (184.3–280.5) µmol/L and 245.0 µmol/L, respectively. The median plasma BCAAs levels for living unrelated individuals appeared to be maintained.

The single deceased donor had higher plasma BCAA levels compared to living related and living unrelated donors after 1-year and 5-year follow-ups. Specifically, isoleucine, leucine, and valine levels were 228.0 and 248.0 µmol/L, 180.0 and 334.0 µmol/L, and 230.0 and 496.0 µmol/L, respectively.

Median total bilirubin was normal among living related (7.0 mg/L, IQR 5.1–7.9) and living unrelated donors (8.75 mg/L, IQR 6.5–16.4) compared to a single deceased donor (16.0 mg/L). Albumin, ALT, AST, and GGT levels were consistently within normal ranges across all groups. These descriptive outcomes indicate relatively stable graft function in related and unrelated donor recipients, though variability in single deceased donor recipients warrants further research.

Reference normal ranges: Isoleucine (50–150 µmol/L), Leucine (100–200 µmol/L), Valine (150–300 µmol/L), Alanine aminotransferase (<40 U/L), Aspartate aminotransferase (<40 U/L), Gamma-glutamyl transferase (<60 U/L), Albumin (35–50 g/L), Bilirubin (<12 mg/L).

## Discussion

Leveraging LT procedures performed for metabolic disorders between 2015 and 2023 on ten patients at the King Fahad Specialist Hospital-Dammam, this retrospective study demonstrates that LT is a highly effective treatment for classical MSUD, providing excellent graft survival, significant metabolic improvement, and liberation from a protein-restricted diet. All ten patients undergoing the MSUD procedure revealed that, irrespective of the donor type, excellent outcomes were achieved with no significant clinical or laboratory differences observed between living-related, living-unrelated, or deceased donor recipients, as observed through the comparable post-transplant BCAA levels and graft function across groups. For instance, the median 1-year post-LT leucine level was 164 µmol/L in the living-related group vs. 175.5 µmol/L in the living-unrelated group, both representing a dramatic reduction from pre-transplant medians (713 and 253.5 µmol/L, respectively) and bringing the majority of patients close to the normal range (100–200 µmol/L). Furthermore, graft function remained stable, with median ALT levels well within the standard limit (<40 U/L) for all donor groups (Living-Related: 20.0 U/L; Living-Unrelated: 31.5 U/L; Deceased: 29.0 U/L).

In regions where there is a critical shortage of deceased donor organs, this observation has profound implications. This paper posits that the primary challenge in managing MSUD lies in the prevention of metabolic crises through dietary control, aligning with Hassan & Gupta's definition of MSUD as an autosomal recessive metabolic disorder characterized by a deficiency in the BCKDH enzyme complex, which is necessary for the breakdown of the BCAAs isoleucine, leucine, and valine (3). In this cohort, indications for LT evolved beyond recurrent metabolic crises to include broader quality-of-life issues and, notably, the pragmatic challenge of formula unavailability, a problem exacerbated globally during the COVID-19 pandemic as observed by Marino et al. (6). Thus, in line with recommendations articulated by Olson & Berger, this paper highlights that early diagnosis through the national newborn screening program allows for prompt dietary intervention, additionally, this screening also allows for the identification of patients who may benefit from definitive therapy with LT before the incidence of irreversible neurological damage occurs (7).

One of the key findings and a key area of consideration realized from this retrospective analysis is the metabolic sufficiency of a liver graft from a heterozygous, related living donor, which is curative despite the extrahepatic expression of the BCKDH enzyme complex. This data contributes to the growing body of evidence that a graft from a heterozygous carrier provides sufficient enzymatic activity to maintain metabolic control and prevent crises under most circumstances (8–12). The median BCAA levels in our living-related donor group, while occasionally above the normal range, were substantially lower than pre-transplant levels and clinically stable, allowing for a regular diet aligning with previous reports of successful transplantation from heterozygous parents (13, 14). For instance, Feier et al. similarly found no significant difference in metabolic control or graft survival between living-related and deceased donor recipients (15). However, it is prudent to counsel families that the residual risk of metabolic decompensation during severe systemic illness may be marginally higher than with a wild-type graft, warranting vigilance and prompt consultation with a metabolic specialist if concerning symptoms arise (2).

The overall outcomes in our cohort were excellent, with 100% patient and graft survival and no donor complications. The high rate of domino transplantation utilizing the explanted livers further underscores the dual benefit of LT for MSUD, curing the recipient while providing a life-saving graft for another patient. The universally reported satisfaction among families is a robust outcome measure, strongly linked to the dramatic improvement in quality of life, particularly the ability to consume a regular diet in a culture where food is central to social life.

### Limitations

This retrospective analysis highlights several limitations realized in the research process. Firstly, the study's retrospective design and small sample size are key areas of limitation, notably, the small number of participants in the deceased donor group, limiting the statistical power necessary to detect subtle differences between different donor types. Moreover, the median follow-up of 3 years is relatively short for assessing long-term graft function and metabolic stability. Furthermore, we lacked a systematic, objective assessment of neurocognitive outcomes and quality of life, relying instead on subjective reports of satisfaction. The absence of standardized psychological or developmental metrics means that the full impact of LT on neurological trajectory and psychosocial well-being could not be quantitatively evaluated. Future prospective studies with larger cohorts, longer follow-up, and validated neuropsychological and quality-of-life instruments are needed to confirm these findings.

## Conclusions

LT stands as a transformative intervention for MSUD, effectively replacing a life of relentless dietary vigilance and metabolic uncertainty with one of stability and remarkable dietary freedom. Our experience confirms that this procedure offers a definitive cure, with outstanding survival rates and the profound benefit of a regular diet, fundamentally improving the quality of life for patients and their families. Critically, our findings demonstrate that in environments where deceased donor organs are a scarce resource, living-related donation, including from heterozygous parents, emerges as a robust and viable pathway. These grafts provide comparable metabolic control and long-term outcomes, opening a crucial avenue for treatment. This strategy successfully expands the donor pool, ensuring that more children can access this life-changing therapy and experience a childhood unburdened by the strictures of MSUD.

## Data Availability

The original contributions presented in the study are included in the article/Supplementary Material, further inquiries can be directed to the corresponding author.
